# Decomposition-Based Multi-Step Forecasting Model for the Environmental Variables of Rabbit Houses

**DOI:** 10.3390/ani13030546

**Published:** 2023-02-03

**Authors:** Ronghua Ji, Shanyi Shi, Zhongying Liu, Zhonghong Wu

**Affiliations:** 1 College of Information and Electrical Engineering, China Agricultural University, Beijing 100083, China; 2 State Key Laboratory of Animal Nutrition, College of Animal Science and Technology, China Agricultural University, Beijing 100193, China

**Keywords:** correlated time series, multivariate multi-step prediction, deep learning algorithm, environment variables forecasting

## Abstract

**Simple Summary:**

Forecasting rabbit house environmental variables is critical to achieving intensive rabbit breeding and rabbit house environmental regulation. As a result, this paper proposes a decomposition-based multi-step forecasting model for rabbit houses using a time series decomposition algorithm and a deep learning combinatorial model. The experimental results demonstrated that the proposed method could provide accurate decisions for rabbit house environmental regulation.

**Abstract:**

To improve prediction accuracy and provide sufficient time to control decision-making, a decomposition-based multi-step forecasting model for rabbit house environmental variables is proposed. Traditional forecasting methods for rabbit house environmental parameters perform poorly because the coupling relationship between sequences is ignored. Using the STL algorithm, the proposed model first decomposes the non-stationary time series into trend, seasonal, and residual components and then predicts separately based on the characteristics of each component. LSTM and Informer are used to predict the trend and residual components, respectively. The aforementioned two predicted values are added together with the seasonal component to obtain the final predicted value. The most important environmental variables in a rabbit house are temperature, humidity, and carbon dioxide concentration. The experimental results show that the encoder and decoder input sequence lengths in the Informer model have a significant impact on the model’s performance. The rabbit house environment’s multivariate correlation time series can be effectively predicted in a multi-input and single-output mode. The temperature and humidity prediction improved significantly, but the carbon dioxide concentration did not. Because of the effective extraction of the coupling relationship among the correlated time series, the proposed model can perfectly perform multivariate multi-step prediction of non-stationary time series.

## 1. Introduction

The future development trend of the rabbit breeding industry is toward intensification. The precise regulation of rabbit house environmental variables is the premise of intensive breeding, and the ability to accurately predict rabbit house environmental variables is the foundation for achieving environmental regulation.

Temperature, relative humidity, and carbon dioxide concentration are the most important environmental variables in a rabbit house. The prediction of rabbit house environmental variables falls under the category of time series prediction. Time series forecasting algorithms are classified into two types based on implementation theory: traditional mathematical algorithms [1,2] and machine learning algorithms [3,4]. To effectively predict the environmental parameters of livestock houses, researchers have proposed Elanco ammonia concentration prediction equation [5] for cattle houses and ammonia concentration mass balance model [6] for swine houses using traditional mathematical forecasting algorithms. However, the prediction methods based on mathematical principles have poor generalization performance and low stability. Machine learning algorithms are classified as either machine learning or deep learning algorithms [7,8]. Researchers have proposed using models such as Support Vector Regression (SVR) [9,10] for machine learning algorithms to predict livestock house environmental variables. These models can accurately predict non-stationary single-parameter time series data. The variables in the rabbit house environment are coupled, and the machine learning model’s relatively simple structure makes fully exploring the coupling relationship between the variables difficult. With the advent of deep learning, researchers have used Long Short Term Memory (LSTM) [11], Back Propagation Network (BPN) [12], and other models to predict the gas concentration of the chicken house, as well as the temperature and humidity of the pig house. Deep learning algorithms can mine the complex characteristics of variables more effectively.

According to the forecasting step, time series forecasting algorithms are classified as single-step forecasting or multi-step forecasting. Single-step time series forecasting algorithms, such as Elman neural network [13] and SVR [14], have produced relatively accurate results; however, in rabbit house scenarios, environmental regulation is usually delayed by a small amount of time [15], so single-step forecasting algorithms do not meet the time requirement. Nonetheless, multi-step forecasting algorithms such as LSTM-based deformation models [16,17], SVR-based models [18], and Echo State Network-based models [19] can be used to predict rabbit house environmental variables in multiple steps.

Based on the number of model output variables, time series forecasting algorithms are further classified as univariate forecasting algorithms and multivariate forecasting algorithms [20,21]. The series of rabbit house environmental variables exhibits both periodicity and nonlinearity [22] and mutual coupling between variables [23]. The prediction accuracy will suffer if the prediction is solely based on the law of the predicted variable and ignores the impact of other variables. Therefore, in rabbit house environmental variable time series prediction, multivariate and multi-step forecasting corresponds to the actual demand. Researchers have proposed a few prediction models based on Graph Neural Network [24], Recurrent Neural Network [25], and Echo State Network [26,27] that improved prediction accuracy by effectively extrapolating and fully utilizing the coupling relationship between multiple variables, which provides a reference for the multivariate and multi-step prediction of rabbit house environmental variables, as deep learning algorithms have rapidly developed.

The multivariate and multi-step time series forecasting model’s input–output mapping is quite complex and has a low prediction accuracy. To effectively carry out long sequence prediction by fully mining the dependence relationship between time series, researchers have proposed various models based on mathematical transformations [28], Temporal Convolutional Network [29], and Ensemble Empirical Mode Decomposition [30]. Informer [31], a long series forecasting model based on the attention mechanism proposed in 2021, effectively extracts the coupling relationship between the correlated time series through the complex nonlinear mapping relationship established by the encoder and decoder, reduces computational complexity by using the sparse self-attention mechanism, and predicts the correlated time series accurately and efficiently. Using a sparse self-attention mechanism reduces computational complexity, allowing for more accurate and efficient prediction of correlated long-time series.

To ensure the algorithm’s prediction accuracy, it is critical to allow sufficient time for control of decision-making in practical situations. This paper proposes a decomposition-based multi-step forecasting model for non-stationary correlated time series to address this issue.

To improve the prediction accuracy of rabbit house environmental variables and provide sufficient time for control of decision-making, this paper proposes to establish a decomposition-based multi-step forecasting model for rabbit house environmental variables based on previous research results and to provide an effective reference for rabbit house environmental supervision decision-making.

## 2. Materials and Methods

### 2.1. Time Series Forecasting Algorithms

#### 2.1.1. Long Short Term Memory

**Figure 1:**
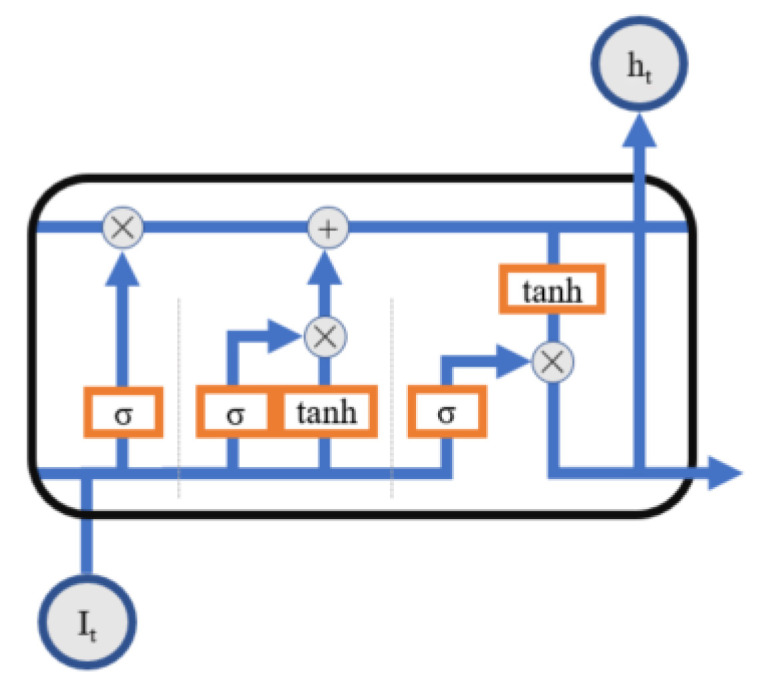
Structure graph of short and long time memory network unit.

LSTM is a time series prediction model that can effectively avoid gradient disappearance and gradient explosion. The trend component obtained by STL decomposition is the long-term change trend of this variable, with stable fluctuation and a small standard deviation, which can be accurately predicted using a simple structure time series prediction model. To that end, LSTM is used in this study to predict the trend component of decomposed time series data. Figure 1 depicts the LSTM cell structure.

As shown in Figure 1, for a given input sequence I$=\left\{I_{1},\;I_{2},\;\ldots ,I_{n}\right\}$, LSTM time series data prediction process is mainly divided into three steps. In the first forgetting step, the LSTM unit receives the input data $h_{t-1}$ of the hidden layer unit at the previous time step and the input data $I_{t}$ at the current time step for weighted sum calculation to control which input of the previous time step needs to be left over. The data obtained in the previous step are fed into the sigmoid function in the second step to obtain the $f_{t}$, which is the Information that must be forgotten. Furthermore, the weighted sum of $h_{t-1}$ and $x_{t}$ is calculated. The output step is the third step. The second stage data are fed into the sigmoid and tanh functions to obtain the input gate value and state information. The cell state is updated based on the most recent time data. The value of the output gate is determined to obtain the output data $O_{t},$ and the current hidden layer unit value $H_{t}$ is later taken to the next unit.

#### 2.1.2. Informer

**Figure 2:**
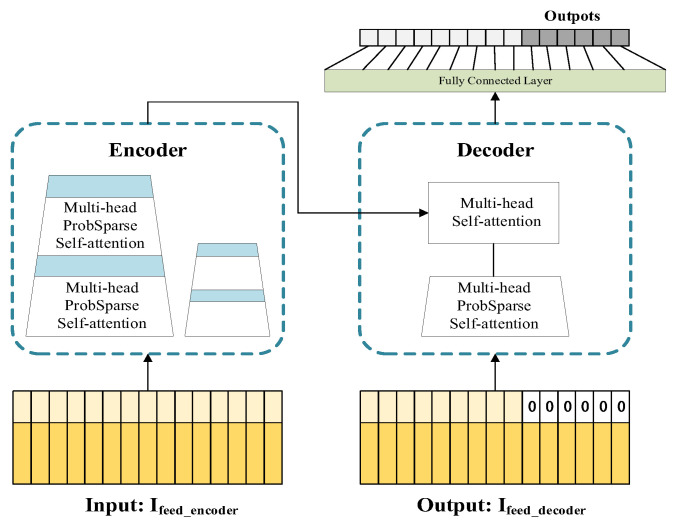
Informer structure diagram.

The Informer uses the Encoder-Decoder architecture, whose overall structure is shown in Figure 2. For the time series sample pair $\left\{I_{j},I_{j+1},\ldots ,I_{j+s-1}\right\}$ $\left\{I_{j+s-l},\ldots ,I_{j+s-1+p}\right\}$, the encoder receives a long sequence $\left\{I_{j},I_{j+1},\ldots ,I_{j+s-1}\right\}$ as input, through the sparse self-attention module, combined with the self-attention distillation mechanism. The feature vector $V_{e}$ is obtained. $\left\{I_{j+s-l},\ldots ,I_{j+s-1+p}\right\}$ through mask processing, and the feature vector $V_{d}\;\mathrm{is}\;\mathrm{obtained}\;\mathrm{throught}\;\mathrm{the}\;$input to the sparse self-attention module of the decoder. The query vector is calculated by $V_{e}$, then the key vector and the value vectors are calculated by $V_{d}$. Finally, the full attention mechanism and the fully connected layer are used to obtain the output, where s, l, and p denote the three custom variables seq_len, label_len, and pred_len for data processing, respectively.

### 2.2. Model Structure

**Figure 3:**
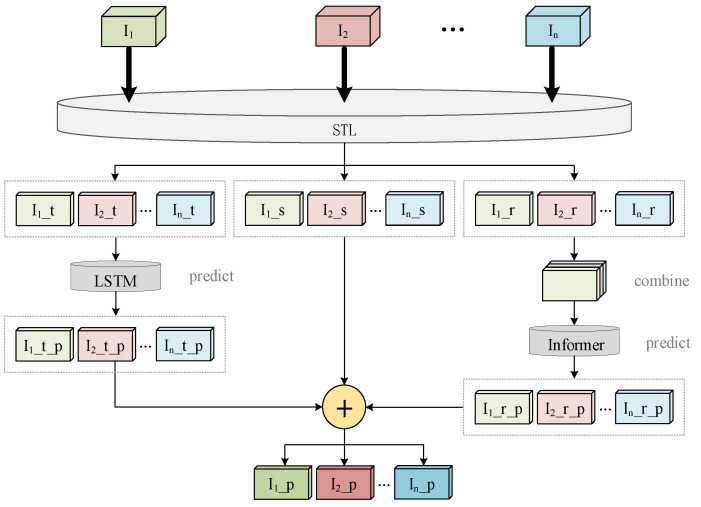
The fundamental structure of the decomposition-based multi-step forecasting model for the environmental variables of rabbit house.

A non-stationary series is the rabbit house variates time series. Non-stationary series are typically divided into three components: trend, seasonal, and residual, and each data component has distinct characteristics. It was discovered that decomposing the non-stationary time series first and then predicting each part individually improve prediction operation accuracy significantly. Figure 3 depicts the basic structure of the decomposition-based multi-step forecasting model for rabbit house environmental variables.

The decomposition-based multi-step forecasting model for rabbit house environmental variables includes three primary stages, as shown in Figure 3.

The first stage is to decompose the time series into three parts. The input of model is the correlated time series $\left\{I_{1},I_{2},\ldots ,I_{n}\right\}$. The time series is decomposed into a trend sequence$\left\{I_{1\_t},I_{2\_t},\ldots ,I_{n\_t}\right\}$, the seasonal sequence $\left\{I_{1\_s},I_{2\_s},\ldots ,I_{n\_s}\right\},$ and the residual sequence$\left\{I_{1\_r},I_{2\_r},\ldots ,I_{n\_r}\right\}$ by Seasonal and Trend decomposition using Loess (STL), which is a robust and versatile time series decomposition method and is used to decompose time series in this paper.

The second stage entails predicting each of the three components individually. Because the trend component of a time series is relatively stable, the LSTM model is used for prediction. Then the outputs of the LSTM model are the predicted trend component of each series $\left\{I_{1\_t\_p},I_{2\_t\_p},\ldots ,I_{n\_t\_p}\right\}$. The residual component is predicted by the Informer model. The outputs of the Informer model are the predicted residual component $\left\{I_{1\_r\_p},I_{2\_r\_p},\ldots ,I_{n\_r\_p}\right\}$. The seasonal component does not need to be predicted because it is a periodic constant.

The final stage is to obtain the final prediction result, which can be obtained by adding the predicted values of the trend component, residual component, and seasonal component of the correlated time series. The following equation shows how the final forecast value is calculated:

(1) $$I_{i\_p}=I_{i\_t\_p}+I_{i\_s}+I_{i\_r\_p}$$

where $I_{i\_p}$ is the final forecast value of the time series. $I_{i\_t\_p}\;\mathrm{and}\;I_{i\_r\_p}$ are the predicted values of trend component and residual component for the *i_th_* series. $I_{i\_s}$ is the seasonal component of the *i_th_* series.

### 2.3. Data Acquisition

The environmental variables of the rabbit house were collected at Qingdao Kangda Rabbit Co. Ltd. in Shandong Province, China. The company has a large rabbit breeding base. The rabbit house is a 46-m-long enclosed structure with a span of 11.7 m and a height of 4.9 m.

**Figure 4:**
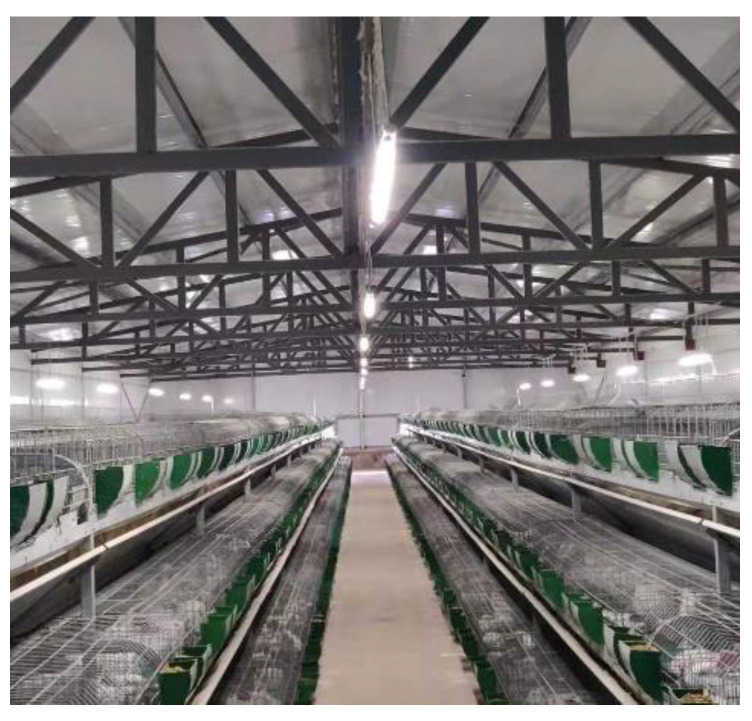
The actual situation inside the rabbit house.

**Figure 5:**
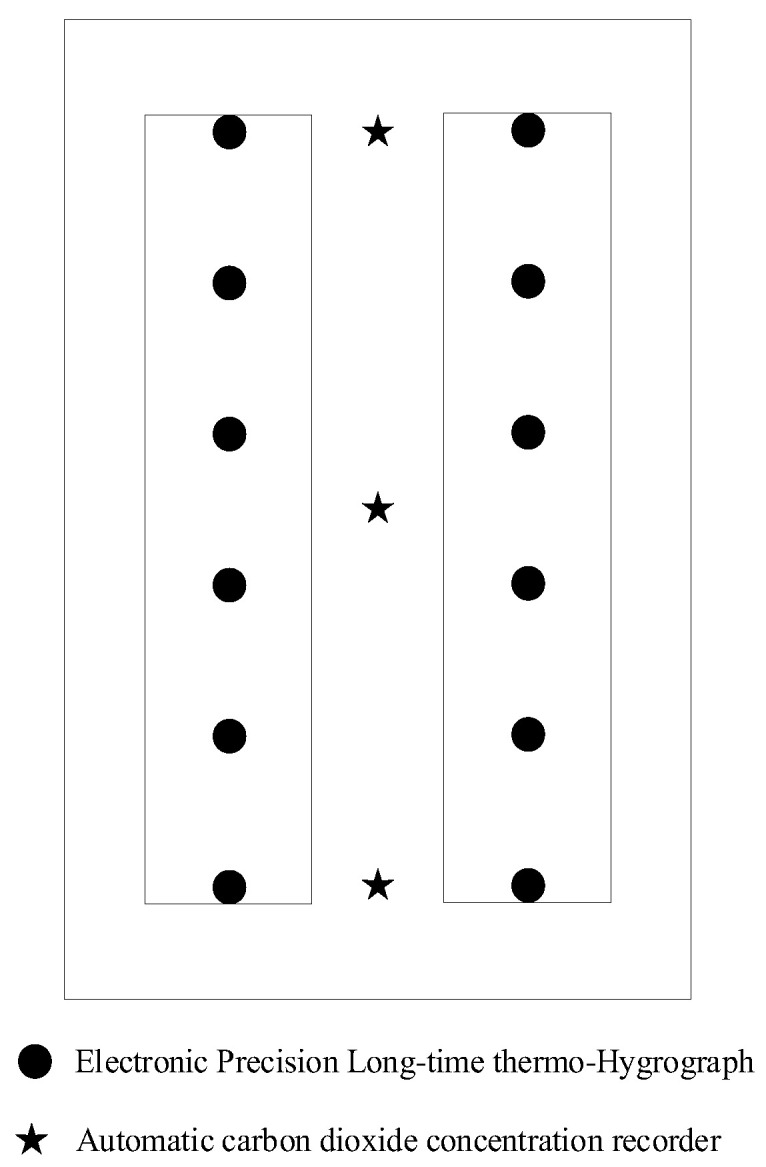
The planar distribution of the sensors inside the rabbit house.

Figure 4 depicts the current situation inside the rabbit house. The environmental variables inside the rabbit house were collected using twelve automated temperature and humidity recorders (Apresy, 179-TH) and three automated carbon dioxide recorders (Tian Jian Hua Yi, EZY-1). Figure 5 depicts the sensors’ planar distribution.

The environmental variables inside the rabbit house were collected at a frequency of 10 min from 00:00 on 30 September 2020 to 23:50 on 28 November 2020. Finally, a total of 8640 rabbit house environmental variables were obtained.

### 2.4. Dataset Preprocessing

#### 2.4.1. Missing Data

Due to the interference of equipment, external factors, and other factors during the acquisition data process of rabbit house environmental variables, the collected data contain some missing values, which is primarily caused by mechanical failures in data collection or storage. Based on the distribution, missing values can be classified as completely random missing or completely non-random missing. The missing data in rabbit house environmental variables are not random. Because the rabbit house’s environmental variables are obtained through continuous timing acquisition every ten minutes, there will be no sudden environmental change. In this study, the mean value of the data before and after the missing value was used to fill in the missing value.

#### 2.4.2. Normalization

The dimensions and dimensional units of the rabbit house environment variables vary. When the model is built directly, it tends to focus on variables with larger dimensions, resulting in low model prediction accuracy and slowing training speed. To eliminate the dimensional effect between the parameters, the data must be standardized so that the preprocessed data is limited to a specific range, typically between zero and one. This method’s calculation formula is as follows:

(2) $$y_{i}=\frac{x_{i}-x_{min}}{x_{max}-x_{min}}$$

where $x_{i}$ and $y_{i}$ are the values before and after normalization, respectively. $x_{min}$ and $x_{max}$ are the minimum and maximum values in the same variables, respectively.

### 2.5. Dataset Analysis

The environmental variables measured inside the rabbit house were processed and analyzed. The results are shown in Table 1.

**Figure 6:**
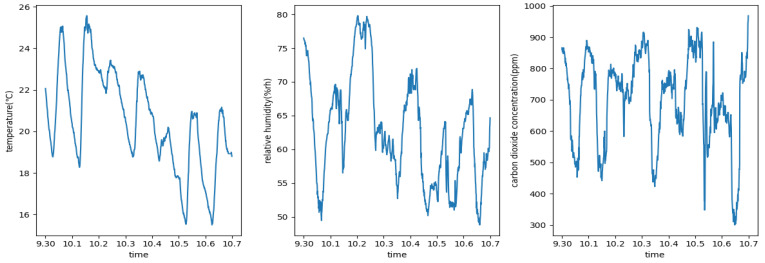
Rabbit house environmental variables within a week.

As shown in Figure 6, three line graphs visualize the fluctuation of the temperature, the relative humidity, and the carbon dioxide concentration variables of the rabbit house within a week.

Table 1 and Figure 6 show that the environmental variables of the rabbit house have a strong periodicity.

To summarize, the three most important environmental variables within the rabbit house fluctuate on a regular basis. The temperature fluctuates in the opposite direction of the relative humidity and carbon dioxide concentration once every 24 h, while the latter two fluctuate in very similar ways.

**Figure 7:**
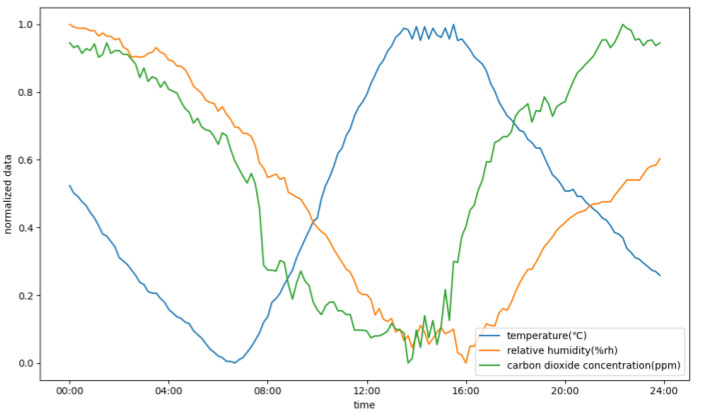
The rabbit house environmental variables within a day.

Figure 7 depicts the normalized temperature, relative humidity, and carbon dioxide concentration in the rabbit house over one day.

The temperature in the rabbit house reaches its lowest point around 7:00 a.m. every day, gradually rises to its peak around 15:00 p.m. every afternoon, and then gradually decreases toward nightfall, as shown in Figure 7. Furthermore, the data show that when the temperature is at its highest, the relative humidity and carbon dioxide concentration are at their lowest, implying that the trend in change of the relative humidity and carbon dioxide concentration is the inverse of that of temperature. Pearson’s correlation coefficient was calculated using the data from day one from the rabbit house’s temperature, relative humidity, and carbon dioxide concentration environment variables. Table 2 summarizes the results of those calculations.

Consequently, three conclusions can be drawn: first, the daily temperature in the rabbit house is strongly correlated with the relative humidity; second, the temperature is correlated with the carbon dioxide concentration; and third, the relative humidity is correlated with carbon dioxide concentration.

The rabbit house time series of environmental variables show periodicity and a strong coupling relationship.

### 2.6. Experimental Settings

The dataset contains a total of 8640 samples representing rabbit house environmental variables. Temperature, relative humidity, and carbon dioxide concentration are all included in each sample. The first 7680 samples are chosen as the training set, and the last 960 consecutive samples are chosen as the test set.

Model parameters have a significant impact on model performance. The following are the proposed model parameters. The LSTM model is made up of three layers: an input layer, a hidden layer, and an output layer. The LSTM model is fed with the temperature, relative humidity, and carbon dioxide concentration trends. The LSTM model’s output is the predicted value of the trend component of temperature, relative humidity, or carbon dioxide concentration. The number of hidden layers is set to four. The Informer model is made up of an encoder and a decoder. The residual component of temperature, relative humidity, and carbon dioxide concentration are the encoder’s inputs, and the predicted value of the inputs is the decoder’s output. The parameters for each module of the Informer model are shown in Table 3.

The training parameters of Informer models are set as shown in Table 4.

## 3. Results and Discussion

### 3.1. Effect of Model Parameter Settings on Prediction Accuracy

The input sequence length of the encoder and decoder modules has a significant impact on model performance in the Informer model. There is the encoder input sequence length, denoted by seq_len, and the decoder input sequence length, denoted by label_len and pred_len. The predicted sequence’s length is label_len, and the model’s output sequence is the last pred_len data in the predicted sequence.

Based on preliminary test results and the experience of relevant breeding experts, we discovered that it takes 20–70 min to adjust the environmental variables in the rabbit house from the initial value to the desired value. The model’s output sequence length should be greater than the maximum regulation time to allow enough time for the rabbit house’s environmental regulation.

The carbon dioxide concentration in the rabbit house varies greatly over time and is difficult to predict. Therefore, in the experiment of selecting the sequence length of the informer model, the carbon dioxide concentration was chosen as the model output. The Dataset_ KD_ minute class has been designed to load, segment, and normalize the dataset recorded to ensure that the environment variables of the rabbit house are fit for the Informer model. Dataset_ KD_ minute class divides the data into sequence data and time stamp data. Time stamped data are the corresponding time stamp of the sequence data, which is transformed into a vector, and extended to the same dimension as the sequence data vector through the neural network, and superimposed with the sequence data vector as the final input data.

The sequence length of the Informer model is set as follows: seq_len is set as 24 (4 h), 36 (6 h), 48 (8 h), 72 (12 h), and 84 (14 h) separately. label_len is set n-24 and n-12 separately, where *n* is the corresponding seq_len value; pred_len is set as 7 (70 min), 9 (90 min), and 12 (120 min) separately. The experiments were carried out separately using the above model parameters, which were then combined. Table 5 displays the experimental results.

It can be found that MSE decreases with an increase in pred_len when seq_len and label_len are the same. Considering that pred_len is stable, the MSE decreases while seq_len increases. The reason for this is that as the input sequence lengthens, the correlation between the input and output sequences weakens. The best prediction is generated when seq_len is set to 24, label_len is set to 12, and pred_len is set to 7.

### 3.2. Effect of Sequence Decomposition on Model Prediction

In this section, the experiment was carried out to investigate if the model can accurately predict the environmental parameters of the rabbit house. The goal of this experiment is to evaluate if sequence decomposition improves prediction accuracy, or in other words, to investigate if the proposed model can accurately predict the environmental variables of the rabbit house.

**Figure 8:**
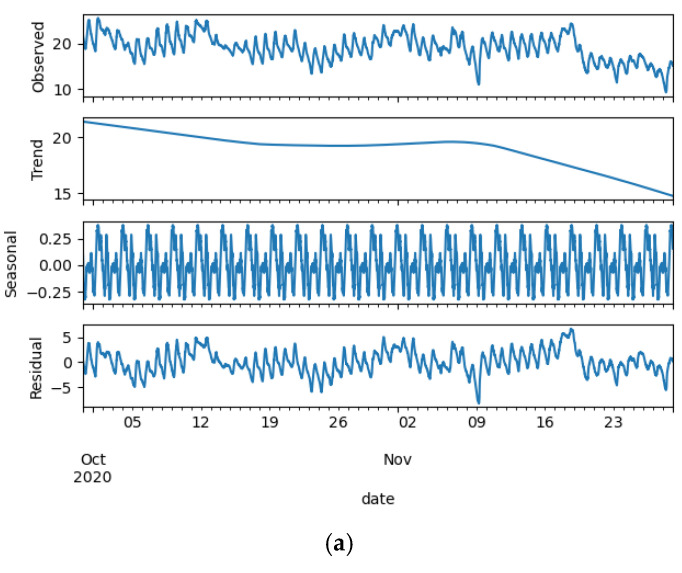

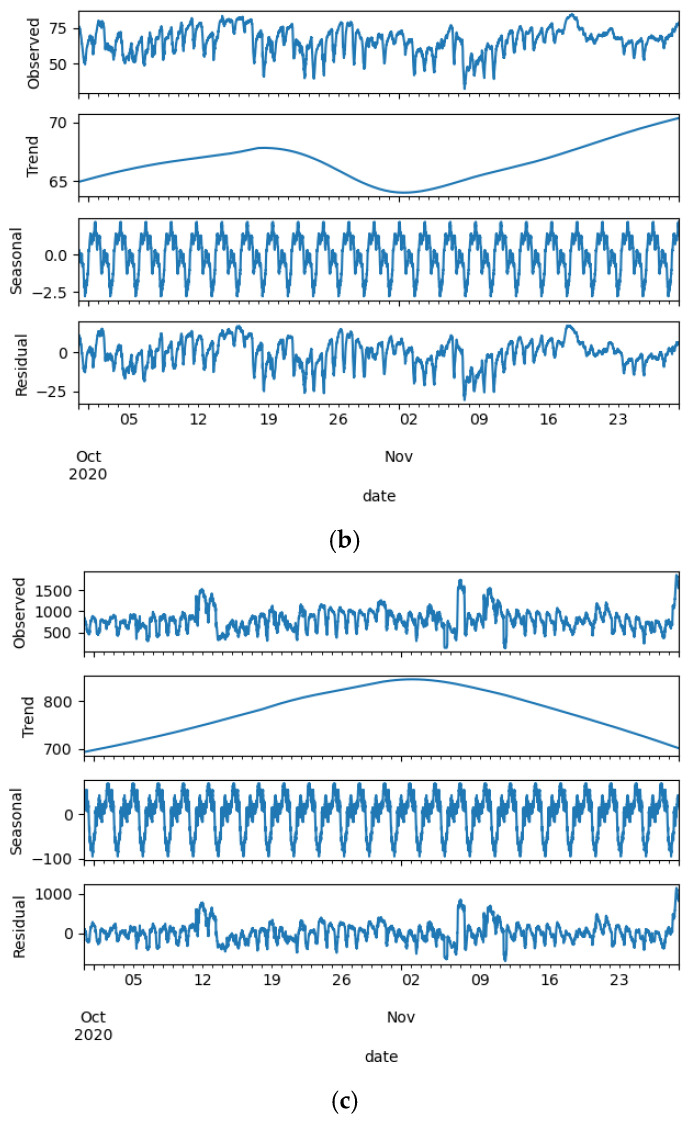
The decomposition results for the rabbit house environmental parameters. (**a**) Temperature decomposition diagram, (**b**) Humidity decomposition diagram, (**c**) Concentrations of carbon dioxide decomposition diagram.

The STL algorithm is primarily used to decompose the environmental variables, and the decomposition results are shown in Figure 8.

The change trend of the residual component environmental variables of the rabbit house is found to be similar to that of the original variables. Table 6 shows the results of calculating the correlation coefficient among the residual component environmental variables of the rabbit house.

t_r, h_r, and c_r in Table 6 are the residual component environmental variables of the rabbit house, which were decomposed by the STL algorithm. After decomposition, the correlation between the residual components of the rabbit house’s environmental variables improves in comparison to Table 2. The coefficient of correlation between residual temperature and relative humidity has shifted slightly. Besides, the carbon dioxide concentration residual component with the temperature residual component or with the relative humidity residual component, the correlation coefficient increased by 24.76% or by 10.65%, respectively.

To test whether the proposed model can predict the correlation time series, the Informer, SVR, XGBoost, and the proposed model were used to predict the environmental variables of the rabbit house. SVR is a supervised machine learning model that seeks the best-fitting equation. It is capable of predicting time series data. SVR has high prediction accuracy as well as good robustness and generalization ability. XGBoost is a boosting-based supervised learning model. A CART decision tree is generated in each iteration to fit the difference between the sum of the predicted results of all previous trees and the true value. It has the benefits of not overtraining and quick training speed. Table 7 displays the results of the comparison experiments.

As shown above, when the proposed model predicts temperature and humidity, the model evaluation indicators MSE, MAE, and RMSE increase by more than 90% when compared to other models. Namely, when predicting temperature and humidity, the prediction effect of the rabbit house time series can be significantly improved. When used to predict carbon dioxide concentration, the proposed model’s prediction accuracy is less improved. This is because the fluctuation of carbon dioxide concentration in the rabbit house is much greater than the fluctuation of temperature and humidity, indicating that the proposed model is better suited for time series prediction with small fluctuations. Communication with breeders about the cause of the large variation in carbon dioxide concentration reveals that carbon dioxide concentration is affected not only by temperature and relative humidity but also by external factors such as rearing methods, resulting in high expectations for the model’s generalization ability. However, in terms of current breeding needs, the model’s accuracy can meet the decision-making needs of rabbit house environmental regulation.

### 3.3. Effect of Input Variable Types on Model Performance

We performed several experiments to evaluate how the number of input variable types affected model performance when predicting the correlation time series. The experiments were carried out as follows. The proposed model takes one type, two types, and three types as input and outputs a single variable. For example, the model inputs are set to temperature; temperature and relative humidity; temperature and CO_2_ concentration; temperature, relative humidity, and CO_2_ concentration; temperature, relative humidity, and CO_2_ concentration; temperature, relative humidity, and CO_2_ concentration. In contrast, the proposed model is used to predict temperature, and the model prediction accuracy, including MAE, MSE, and RMSE, is recorded.

**Figure 9:**
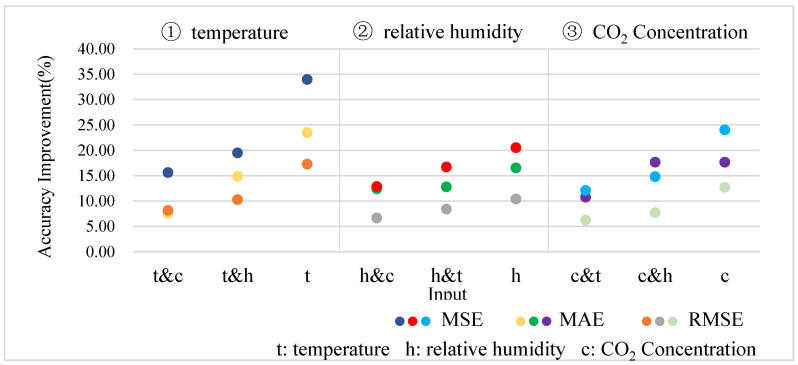
The prediction accuracy improvement with different input.

The results show that the best prediction performance is obtained when the model’s input variable type is three. The percentage improvement in model prediction accuracy was calculated when the model input was three types versus one type and two types. Figure 9 depicts the outcomes. The three areas in Figure 9 represent the model output as temperature, relative humidity, and CO_2_ concentration, respectively. The abscissa represents the input type, and the ordinate represents the percentage improvement in model prediction accuracy.

The results show that when temperature, relative humidity, and carbon dioxide concentration are used to predict the environmental variables of the rabbit house, the proposed model has the best prediction effect. This is because the rabbit house’s environmental variables have a strong coupling relationship. Simultaneously, the coupling relationship between the remaining terms of the three variables is highlighted after decomposing the correlation time series, which is more conducive to improving the prediction effect of the proposed model.

## 4. Conclusions

The model performance suffers when we perform multi-step prediction for correlated non-stationary time series of rabbit house environment because we ignore the time series’ coupling relationship. We propose a decomposition-based multi-step forecasting model for rabbit house environmental variables to address this issue.

The proposed model first decomposes the non-steady time series into trend, seasonal, and remainder components using the STL algorithm and then predicts separately based on the characteristics of each component.

Because its main environmental variables (temperature, humidity, and carbon dioxide concentration) correlate with non-stationary time series, the proposed model predicted the rabbit house’s environmental variables to validate its performance. The time series of the rabbit house’s main environmental variables has a strong time correlation and a significant coupling relationship. A number of comparative experiments were also carried out. Accordingly, we reached the following conclusions:

(1) By decomposing the correlation time series and then making specific predictions, the proposed model can realize multi-step prediction for correlated non-stationary time series. The experimental results demonstrated that the proposed model could perform multi-step prediction for the rabbit house’s environmental variables. It can effectively improve temperature and humidity predictions in particular, but not carbon dioxide concentration predictions.
(2) The length of the Informer model’s input and output in the proposed model has a significant impact on the model’s performance. When *seq_len* and *label_len* are the same, MSE decreases with increasing *pred_len* for the rabbit house’s environmental variables prediction. MSE decreases as *seq_len* increases when *pred_len* remains constant. The effect of predicting environmental variables of the rabbit house is the best when *seq_len*, *label_len*, and *pred_len* are set to 24, 12, and 7, respectively.
(3) Temperature, relative humidity, and carbon dioxide concentration were the environmental variables of the rabbit house. The absolute value of Pearson’s correlation coefficient between any of the above two was greater than 0.5, indicating that the time series of environmental variables of the rabbit house not only had their own time correlation but also had a significant coupling relationship with other variables. The model’s complexity and performance are heavily influenced by the types of input and output. When temperature, relative humidity, and carbon dioxide concentration are used as model inputs, and a single parameter is used as model output, the best prediction result for rabbit house environmental variables is obtained. The coupling relationship between the remaining terms of the correlation time series is highlighted further after the time series is decomposed, which improves the model’s prediction performance. When temperature, relative humidity, and carbon dioxide concentration are used as inputs, the proposed model has the best prediction effect for the rabbit house’s environmental variables.

## Figures and Tables

**Table 1:** Statistical information of the rabbit house environment variables.

Variable	Unit	Range	Mean	Standard Deviation
Temperature	°C	[9.3, 25.57]	19.09	2.79
Relative Humidity	%rh	[32.42, 84.3]	66.03	8.86
CO_2_ Concentration	ppm	[138.75, 1858.57]	802.23	250.82

**Table 2:** Person’s Correlation Coefficient for rabbit house environmental variables.

	Temperature	Relative Humidity	CO_2_ Concentration
Temperature	1	−0.8276	−0.5149
Relative Humidity	−0.8276	1	0.6505
CO_2_ Concentration	−0.5149	0.6505	1

**Table 3:** The Parameters of the Informer model.

Main Modules	Sub-Model	Parameters	Type/Value
Encoder	Sparse Self-Attention Module	Number	1
		Number of heads	8
		Number of units	5
	Self-attentive distillation module	Number of stack levels	3,2,1
Decoder	Attention Mechanisms Module	Number of stories	1
		Type	Full attention
	Fully connected layer	Number of stories	1
		Number of hidden units	512

**Table 4:** The Training parameters for the Informer model.

Parameters	Value/Type
Optimizer	Adam
Epoch_size	6
Batch_size	96
Loss Function	MSE
Initial Learning Rate	0.001
Learning rate adjustment method	Each epoch goes down by half
Iteration times	6
Drop_out	0.05

**Table 5:** Effect of model parameters on prediction accuracy of the model.

seq_len	label_len	pred_len	MSE (ppm)
24	0	7	0.05255134
		9	0.05477738
		12	0.082935
	12	7	0.04983531
		9	0.0659451
		12	0.08847401
36	12	7	0.0786688
		9	0.07736252
		12	0.11616067
	24	7	0.05427639
		9	0.06950006
		12	0.09658156
48	24	7	0.08289333
		9	0.0991382
		12	0.14262368
	36	7	0.07137094
		9	0.08005989
		12	0.12634541
72	48	7	0.09766254
		9	0.13091703
		12	0.15992408
	60	7	0.0786742
		9	0.13212924
		12	0.15188387
84	60	7	0.09868335
		9	0.1437092
		12	0.16232075
	72	7	0.08037249
		9	0.13032459
		12	0.15770504

**Table 6:** Correlation coefficient of the residual component environmental variables of the rabbit house.

Variable	t_r	h_r	cd_r
t_r	1	−0.8277	−0.6424
h_r	−0.8277	1	0.7198
cd_r	−0.6424	0.7198	1

**Table 7:** The prediction results of two models for the prediction of correlated time series.

Predicted Variable	Model	MAE	MSE	RMSE
Temperature	Informer	0.12279760	0.02539201	0.15934873
	SVR	0.16925226	0.02864633	0.10879960
	XGBoost	0.13623399	0.02993292	0.17301134
	Proposed model	0.01167244	0.00030404	0.01743695
Humidity	Informer	0.12256409	0.02699366	0.16429749
	SVR	0.11360447	0.04006461	0.20016146
	XGBoost	0.13044998	0.02799125	0.16730587
	Proposed model	0.00261323	0.00015509	0.01245363
CO_2_ Concentration	Informer	0.15820476	0.04983531	0.22323823
	SVR	0.14900408	0.05078254	0.22534983
	XGBoost	0.16991315	0.05427113	0.23296165
	Proposed model	0.15369803	0.04048497	0.20120878

## Data Availability

If anyone needs the data used in this study, please contact: jessic1212@cau.edu.cn.
